# The 160 bp Insertion in the Promoter of *Rht-B1i* Plays a Vital Role in Increasing Wheat Height

**DOI:** 10.3389/fpls.2016.00307

**Published:** 2016-03-16

**Authors:** Xueyuan Lou, Xin Li, Aixia Li, Mingyu Pu, Muhammad Shoaib, Dongcheng Liu, Jiazhu Sun, Aimin Zhang, Wenlong Yang

**Affiliations:** ^1^The State Key Laboratory of Plant Cell and Chromosome Engineering, Institute of Genetics and Developmental Biology, Chinese Academy of SciencesBeijing, China; ^2^Graduate University of Chinese Academy of SciencesBeijing, China; ^3^The Collaborative Innovation Center for Grain Crops in Henan, Henan Agricultural UniversityZhengzhou, China

**Keywords:** *Triticum aestivum* L., *Rht-B1i*, height-increasing effect, promoter, 160 bp insertion

## Abstract

The extensive use of two alleles (*Rht-B1b* and *Rht-D1b*) at the *Rht-1* locus in wheat allowed dramatic increases in yields, triggering the so-called “Green Revolution.” Here, we found that a new natural allelic variation (*Rht-B1i*) containing a single missense SNP (A614G) in the coding region significantly increased plant height against the genetic background of both *Rht-D1a* (11.68%) and *Rht-D1b* (7.89%). To elucidate the molecular mechanism of *Rht-B1i*, we investigated the promoter region. Sequence analysis showed that the *Rht-B1i* promoter could be divided into two classes depending on the presence or absence of a specific 160 bp insertion: *Rht-B1i-1* (with the 160 bp insertion) and *Rht-B1i-2* (without the 160 bp insertion). The promoter of *Rht-B1i-1* contained 32 more possible *cis*-acting elements than *Rht-B1a*, including a unique auxin response element AUXREPSIAA4. Quantitative RT-PCR analysis indicated that the 160 bp insertion is likely to promote the transcription of the *Rht-B1i-1* gene. The coleoptile lengths of wheat varieties treated with IAA, GA_3_, and IAA/GA_3_, combined with the histochemical staining of transgenic *Arabidopsis* containing the *Rht-B1i-1* promoter, showed that the height-increasing effect of *Rht-B1i-1* may be due to the synergistic action of IAA and GA_3_. These results augment our understanding of the regulatory mechanisms of *Rht-1* in wheat and provide new genetic resources for wheat improvement.

## Introduction

The decrease in the stem stature of common wheat has resulted in improved lodging resistance and increased harvest index (Gale and Youssefian, 1985; Youssefian et al., 1992; Miralles and Slafer, 1995). As a result, plant height has made a significant contribution to high and stable yields in common wheat (*Triticum aestivum* L.).

Several wheat loci are or were once associated with decreased plant height and these are termed as *Reduced height* (*Rht*). These loci include the three homoeologs of *Rht-1* (*Rht-A1*; *Rht-B1*; *Rht-D1*) and 19 other loci (*Rht4*–*9*; *Rht11*–*23*; McIntosh et al., 2013; Chen et al., 2015). These *Rht* genes are divided into two subgroups, GA-sensitive genes and GA-insensitive genes, depending on their response to exogenous gibberellic acid (GA; Börner et al., 1996; Pestsova et al., 2008; Chen et al., 2015).

Among GA-insensitive semi-dwarfing genes, two genes in Norin 10, originally named *Rht1* and *Rht2*, were re-designated as *Rht-B1b* and *Rht-D1b*, respectively, as they were found to be mutant alleles of the *Rht-1* loci on the short arms of chromosomes 4B and 4D (Gale et al., 1975; Gale and Marshall, 1976; McVittie et al., 1978; Börner et al., 1996). Under near-isogenic genetic backgrounds, *Rht-B1b* and *Rht-D1b* both have the ability to reduce plant height by 15–20% (Gale and Youssefian, 1985; Flintham et al., 1997; Miedaner and Voss, 2008; Blake et al., 2009). In combination, they can reduce plant height by 40% (Flintham et al., 1997). This effect is caused by a nucleotide substitution that creates a premature stop codon, resulting in truncated DELLA proteins in the region responsible for the GA response (Peng et al., 1999). In the 1960s, the introduction of the *Rht-B1b* and *Rht-D1b* alleles in wheat resulted in a great increase in yield during the ‘Green Revolution’ (Hedden, 2003), which abated a major worldwide food shortage.

At the *Rht-B1* and *Rht-D1* loci, five *Rht-B1* alleles (*Rht-B1a–e*) and four *Rht-D1* alleles (*Rht-D1a–d*) have been isolated and characterized in hexaploid wheat (Peng et al., 1999; Pearce et al., 2011; Wu et al., 2011; Li A.X. et al., 2012; Li Y.Y. et al., 2012; Li et al., 2013; Wen et al., 2013). Compared with the wild-type alleles *Rht-B1a* and *Rht-D1a*, the dwarfism conferred by *Rht-B1b, Rht-B1d, Rht-B1e, Rht-D1b*, and *Rht-D1d* is due to a single nucleotide change that introduces a premature stop codon in the DELLA region of encoded proteins (Peng et al., 1999; Pearce et al., 2011; Li A.X. et al., 2012; Li et al., 2013). *Rht-B1c* derived from “Tom Thumb” differs from *Rht-B1a* by one 2 kb *Veju* retrotransposon insertion, three SNPs in the coding region and one 197-bp insertion and four SNPs in the 1-kb upstream sequence (Wen et al., 2013). The intragenic insertion, which results in an in-frame 90-bp insertion in the transcript and a predicted 30-amino acid insertion within the DELLA domain, is primarily responsible for severe dwarfism (Pearce et al., 2011; Wu et al., 2011; Wen et al., 2013). In contrast, as the strongest height-reducing allele in wheat, *Rht-D1c* derived from “Aibian 1” generates a tandem segmental duplication (TSD) of a 1-Mb region, resulting in two copies of *Rht-D1b* (Pearce et al., 2011; Li Y.Y. et al., 2012).

To facilitate the understanding of the diversity of *Rht-1* and the utilization of new resources in breeding programs, Li et al. (2013) previously identified six new *Rht-A1* allelic variations (*Rht-A1b*–*g*), eight new *Rht-B1* allelic variations (*Rht-B1h*–*o*), and six new *Rht-D1* allelic variations (*Rht-D1e*–*j*) in Chinese wheat germplasm by a modified EcoTILLING method. Li et al. (2013) found that *Rht-B1i* (E205G) have frequencies of 4.2% in Chinese wheat MCC and 2.28% in 1,537 important Chinese wheat cultivars and germplasms, respectively. These frequencies are relatively high among the 10 *Rht-B1* allelic variations (*Rht-B1a, b, h*–*o*). Recently, Wilhelm et al. (2013b) discovered new haplotypes of *Rht-1* in western wheat cultivars and tetraploid and diploid wheat, and found that three haplotypes (*Rht-B1a_2, 3, 4*) also contained an E205G substitution in the poly S/T/V region. Moreover, there are five *Rht-B1a_2*, one *Rht-B1a_3*, and two *Rht-B1a_4* in the 66 bread wheat accessions (nine accessions for bread wheat set 1, 12 accessions for a subset of BW1 accessions widely grown in the UK, and 45 accessions from the INRA worldwide bread wheat core collection of 372 accessions), the frequency of E205G substitution is 12.12%. Sequence alignment revealed that the open reading frame (ORF) sequences of the three haplotypes (*Rht-B1a_2, 3, 4*) in Wilhelm et al. (2013b) is identical to *Rht-B1i* in Li et al. (2013), indicating that *Rht-B1i* widely exist in the Chinese and western wheat cultivars.

Interestingly, the new natural allelic variation *Rht-B1i*, which contains a single missense SNP (A614G) in the coding region resulting in E205G, may increase plant height (Li et al., 2013). However, the underlying mechanism for this effect is unclear. Current studies mainly focus on alleles with dwarfing effects. Moderate dwarfing can increase grain yield in wheat breeding, but excessive dwarfing reduces production owing to decreased biomass. Reports of alleles with height-increasing effects are extremely rare, and elucidating this effect of *Rht-B1i* may enable the allele to contribute to ameliorating excessive dwarfism in wheat. In this study, we isolated and characterized the promoter of *Rht-B1i*. According to the presence or absence of 160 bp insertion, *Rht-B1i* could be divided into *Rht-B1i-1* (with 160 bp insertion in promoter) and *Rht-B1i-2* (without 160 bp insertion in promoter) using molecular markers and sequencing analysis. Quantitative RT-PCR analysis revealed that the 160 bp insertion may promote the transcription of the *Rht-B1i-1* gene. The coleoptile lengths of wheat varieties treated with IAA, GA_3_, and IAA/GA_3_, combined with the histochemical staining of transgenic *Arabidopsis* containing the *Rht-B1i-1* promoter, indicated that the height-increasing effect of *Rht-B1i-1* may be due to the synergistic action of IAA and GA_3_.

## Materials and Methods

### Plant Height Determinations

The Chinese wheat micro-core collections (MCC) consist of Chinese Spring (CS), 155 landraces, 89 Chinese wheat-bred cultivars, and 17 introduced foreign accessions. The MCC is representative of 1% of the national wheat collection but over 70% of the genetic diversity (Wang et al., 2008; Li et al., 2013). We planted accessions containing five genotypes (*Rht-B1a, b, h*–*j*) against the genetic background of *Rht-D1a* and *Rht-D1b* in the field site of the Institute of Genetics and Developmental Biology, Chinese Academy of Sciences (Beijing, China) in 2012 and 2013. There are one accession ‘Chinese Spring’ of *B1a*/*D1a*, eleven accessions of *B1b*/*D1a*, eight accessions of *B1h*/*D1a*, 10 accessions of *B1i*/*D1a* (8 *Rht-B1i-1* and 2 *Rht-B1i-2*), 11 accessions of *B1j*/*D1a*, five accessions of *B1a*/*D1b*, five accessions of *B1b*/*D1b*, three accessions of *B1h*/*D1b*, four accessions of *B1i*/*D1b*, and two accessions of *B1j*/*D1b*. Each accession was planted in 2-m two-row plot with 25 cm between rows and 40 seeds per row, and repeated for three times. Plants were managed using standard wheat production management. We measured plant height at 20 days after flowering using 10 randomly selected plants from each plot, and calculated the means and standard deviations of plant height using Microsoft Office Excel (2013). Differences among groups were analyzed with analysis of variance (ANOVA) procedure using SAS software (version 9.1).

### Coleoptile Length Determinations

We chose CS and four varieties containing *Rht-B1i* (three varieties of *Rht-B1i-1* genotype H8, H83, and H215 containing a promoter with the 160 bp insertion as well as one variety of *Rht-B1i-2* genotype H251 containing a promoter without the 160 bp insertion) to assess the effects of IAA and GA_3_ on coleoptile elongation. Seeds were treated with 1% (w/v) H_2_O_2_ in 4°C for 24 h and rinsed three times with sterile deionized water, then cultured individually with ddH_2_O (control), IAA (10 μM), GA_3_ (10 μM), or IAA/GA_3_ (1:1) in a greenhouse under controlled conditions (23°C, dark) for 7 days. We measured coleoptile length for each treatment. Each experiment used 20 plants, and all experiments were carried out in triplicate. We calculated the means and standard deviations of coleoptile length using Microsoft Office Excel (2013). Differences among groups were analyzed with analysis of variance (ANOVA) procedure using SAS software (version 9.1).

### Wheat DNA and RNA Isolation

We grew 70 wheat lines containing different allelic variations at the *Rht-1* locus (Supplementary Table S1), including CS, Mercia-carrying *Rht-B1e*, 68 Chinese wheat MCC varieties (including 23 Chinese wheat landraces, 37 Chinese wheat bread cultivars, and eight introduced foreign accessions), and 35 Chinese wheat-leading cultivars and important germplasm (Supplementary Table S2). All plants were grown in a greenhouse under controlled conditions (23°C, 16:8 photoperiod) for 2 weeks. We collected young leaves for genomic DNA extraction using the hexadecyl trimethyl ammonium bromide (CTAB) method (Aldrich, 1993).

We randomly selected 30 wheat lines from 46 *Rht-B1i* varieties (11 Chinese wheat MCC lines and 35 Chinese wheat-leading cultivars and important germplasm lines). All plants were grown in a greenhouse under controlled conditions (23°C, 16:8 photoperiod) for 2 weeks, and we collected samples of their young leaves to isolate total RNA using the RNeasy Plant Mini Kit (QIAGEN, Germany). Flag leaves and the fourth internodes from four varieties of *Rht-B1i-1* genotype H8, H83, H213, H215, and one variety of *Rht-B1i-2* genotype H251 at the heading stage were also sampled for RNA extraction. CS was used as control variety. The first strand of cDNA was synthesized using FastQuant RT Kit (with gDNase; TIANGEN, Beijing) according to the manufacturer’s instructions.

### Cloning of Promoters from *Rht-1* Allelic Variations

To clone the promoters of the *Rht-1* allelic variations, we designed two primers, PB-CF and PB-CR (Supplementary Table S3), according to the sequence of *Rht-B1a* (FR719732, *Triticum aestivum Rht-B1a* gene for DELLA protein). We performed PCR in 20-μL reaction volumes containing 200 ng DNA template, 0.4 μM forward and reverse primers, 1 U TaKaRa LA Taq (Takara, Biotechnology Co., Ltd, Dalian, China), 1 × GC Buffer I, and 0.4 mM of each dNTP under the following conditions: 94°C for 5 min, followed by 35 cycles of 94°C for 30 s, 58°C for 30 s, and 72°C for 2 min; a final extension was performed at 72°C for 10 min. PCR products were cloned into pGEM-T easy vectors (Promega, Madison, WI, USA), introduced into *Escherichia coli*, and 10 positive independent clones were commercially sequenced. The promoter sequences and their GC characteristics were analyzed using DNAMAN software, and their *cis*-acting elements were predicted using PLACE and PlantCARE.

### Allele-Specific Markers for *Rht-B1i-1*

To facilitate the use of *Rht-B1i* in wheat-breeding programs, we developed two allele-specific PCR markers, B1i-MF1/MR1 and B1i-MF2/MR2 (Supplementary Table S3), based on the 160 bp insertion of the promoter. PCR was performed on an ABI 9700 thermal cycler (Applied Biosystems) as described above. The PCR products were separated on a 2% agarose gel and visualized using a UV spectrometer after ethidium bromide staining.

### Quantitative Real-Time PCR Analysis

We carried out quantitative real-time PCR (qRT-PCR) to determine the transcript level of *Rht-B1i* using the LightCycler 480 system (Roche, Indianapolis, IN, USA) with SYBR Green I Master (Roche, Indianapolis, IN, USA). The gene-specific primers Rht-B1.EF and Rht-B1.ER (Supplementary Table S3) were used for gene expression analysis with the *Ta4045* gene (Paolacci et al., 2009) as a reference. All qRT-PCR experiments were performed independently with three biological and three technical replicates, respectively. We calculated the relative expression of each gene according to the comparative CT method (ΔΔCT; Livak and Schmittgen, 2001) and calculated the means and standard deviations using Microsoft Office Excel (2013). Differences among groups were analyzed with analysis of variance (ANOVA) procedure using SAS software (version 9.1).

### Expression of the GUS Gene Driven by the Promoter of *Rht-B1i*

To construct the plasmid for promoter analysis, we cloned promoters of *Rht-B1a* and *Rht-B1i-1* with the primers PB-*Pst*I-F/PB-*Nco*I-R to introduce *Pst*I and *Nco*I sites (Supplementary Table S3). The amplified fragment was inserted into the pGEM-T vector and recombined with pCAMBIA 3301 (*35S::GUS*) by replacing the *35S* promoter before the *GUS* reporter gene to generate *pB1a::GUS* and *pB1i-1::GUS*. The plant binary vectors were introduced into *Agrobacterium tumefaciens* strain GV3101 using the freezing and thawing method and then transformed into *Arabidopsis* using the flower-dipping method (Clough and Bent, 1998, modified from Bechtold et al., 1993). Transgenic lines were selected on MS (Murashige and Skoog, 1962) plates with PPT (phosphinothricin, 5 mg/L), and more than 20 transgenic lines were obtained. Histochemical detection of GUS activity (Jefferson et al., 1987) was carried out on the T_3_ transgenic plants. T_3_ plants were grown in MS medium with PPT (5 mg/L) for 7 days, then moved to MS medium with GA_3_ (10 μM), IAA (10 μM), or IAA/GA_3_ (1:1) and grown for 5 days. Seedlings were fixed in 90% (v/v) acetone on ice for 10 min, then infiltrated in GUS staining solution [50 mM sodium phosphate buffer, pH 7.0, 10 mM EDTA, 0.01% Triton X-100, 1 mM potassium ferricyanide, 1 mM potassium ferrocyanide, 500 μg/mL 5-bromo-4-chloro-3-indoyl-β-D-glucuronic acid (X-gluc)], and incubated overnight at 37°C. Later, the GUS solution was replaced with 100% (v/v) ethanol at room temperature for 6 h and kept in 70% (v/v) ethanol at 4°C. The stained seedlings were observed and photographed using light microscopy (Olympus SZX16, Olympus Co., Japan).

## Results

### *Rht-B1i* Significantly Increases the Plant Height of Wheat

**FIGURE 1:**
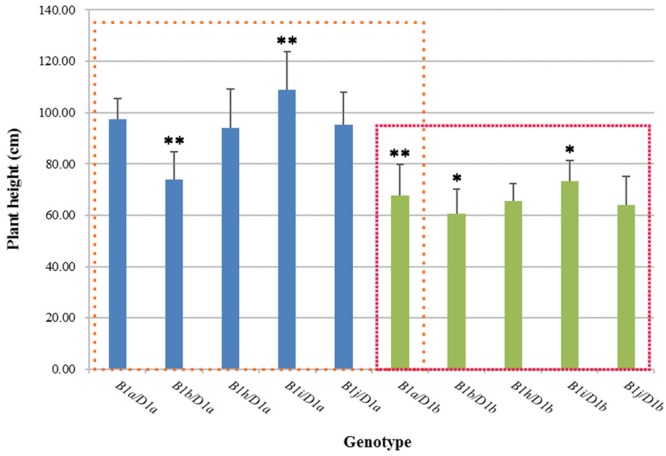
**Plant height in different *Rht-B1* genotypes of wheat.** The blue column represents *Rht-B1* allelic variations against the *Rht-D1a* background, and the green column represents *Rht-B1* allelic variations against the *Rht-D1b* background; the left box compares plant height for different genotypes using *B1a*/*D1a* as a control, and the right box compares plant height for different genotypes using *B1a*/*D1b* as a control. ^∗, ∗∗^Denotes significant differences at 5 and 1% probability levels, respectively.

We analyzed the effects of five genotypes (*Rht-B1a, b, h*–*j*) on wheat plant height against the backgrounds of *Rht-D1a* and *Rht-D1b* using 2 years (2012, 2013) of agronomic trait data (**Figure 1**). Against the background of *Rht-D1a, Rht-B1b* significantly reduced plant height by 24.27% as compared with *Rht-B1a*. The plant heights for *Rht-B1h* and *Rht-B1j* were roughly the same as those for *Rht-B1a*, whereas the height of *Rht-B1i* was significantly increased, by 11.68%, compared with that of *Rht-B1a*. The plant heights for all genotypes were smaller against the background of *Rht-D1b* than against the background of *Rht-D1a*. *Rht-B1b* reduced plant height by 10.91% compared with *Rht-B1a*, whereas *Rht-B1i* increased plant height by 7.89% compared with *Rht-B1a*. These results indicate that *Rht-B1i* significantly increased wheat plant height against both the *Rht-D1a* and *Rht-D1b* backgrounds.

### The Promoter of *Rht-B1i-1* Contains a 160 bp Insertion

To further investigate the height-increasing effects of *Rht-B1i*, we analyzed the entire coding region of *Rht-B1a* and *Rht-B1i*. Sequence alignment showed that the missense SNP occurring in *Rht-B1i* was A614G, giving rise to an E205G amino acid change in the Poly S/T/V region of the encoded DELLA protein (Li et al., 2013). The SIFT (Sorting Intolerant from Tolerant, Sim et al., 2012) score of *Rht-B1i* was 0.41, indicating that the variation had no obvious effect on the protein: non-synonymous SNPs are predicted to be damaging to the encoded protein if the SIFT score is <0.05. Therefore, the height-increasing effect of *Rht-B1i* is unlikely to result directly from the missense mutation in the coding region. Next, we cloned the promoter of *Rht-B1i* using PB-CF/CR primers (Supplementary Table S3). Sequence alignment revealed a 160 bp insertion at 365 bp upstream of the start codon in the promoter of *Rht-B1i*, whereas this insertion was not found in the promoter of *Rht-B1a*.

**FIGURE 2:**
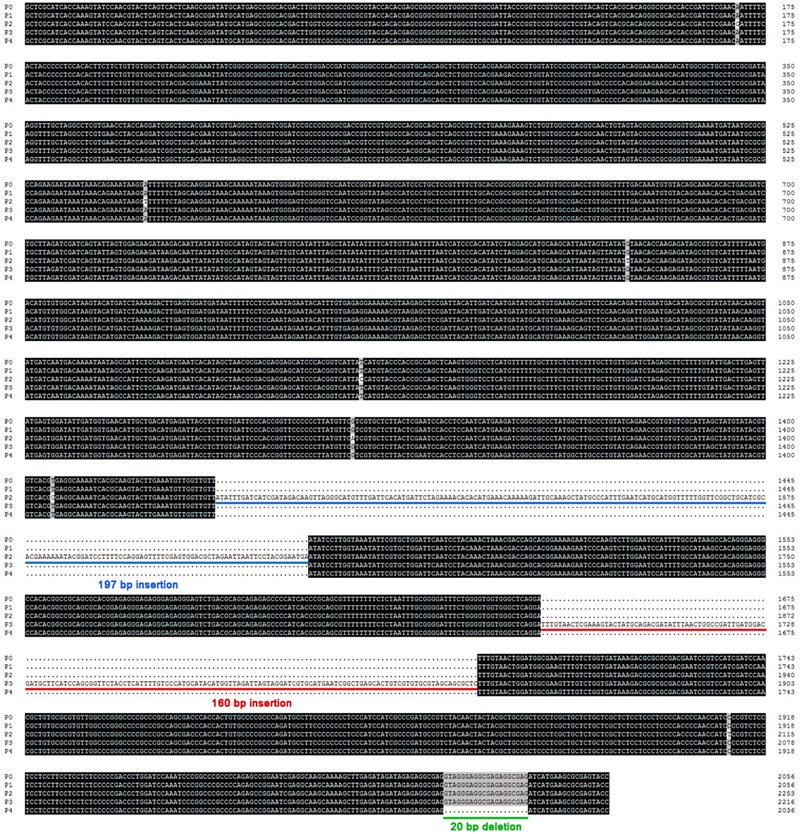
**Sequence alignment for promoters of *Rht-B1* allelic variations in wheat.** Four promoters cloned from seventy wheat lines containing different allelic variations at the *Rht-B1* loci. Compared with the promoter of *Rht-B1a* (P0), P1 was coincide exactly with it, P2 had a 197 bp insertion (sequences on blue lines), P3 had a 160 bp insertion (sequences on red lines), and P4 had a 20 bp deletion (sequences on green line).

To identify whether the 160 bp insertion was specific to *Rht-B1i*, we cloned the promoters of *Rht-B1* allelic variations from *Rht-B1e* (Li A.X. et al., 2012) and 10 other *Rht-B1* allelic variations (*Rht-B1a, b, h*–*o*; Li et al., 2013). The promoters of *Rht-B1a* (P0, 2040 bp) and seven other *Rht-B1* allelic variations (*Rht-B1b, e, j, k, m–o*; P1, 2040 bp, GenBank accession no. LN907868) were identical; the promoter of *Rht-B1h* (P2, 2237 bp, the GenBank accession no. LN907869) contained seven SNPs and a 197 bp insertion at 596 bp upstream of the start codon; the promoter of *Rht-B1i* (P3, 2200 bp, GenBank accession no. LN907870) carried a 160 bp insertion at 365 bp upstream of the start codon; and the promoter of *Rht-B1l* (P4, 2020 bp, GenBank accession no. LN907871) had a 20-bp deletion at 3 bp upstream of the start codon (**Figure 2**, Supplementary Table S1). The 160 bp insertion can only be found in *Rht-B1i*, nevertheless, two of 11 MCC accessions containing *Rht-B1i* have no 160 bp insertion (Supplementary Table S1).

**FIGURE 3:**
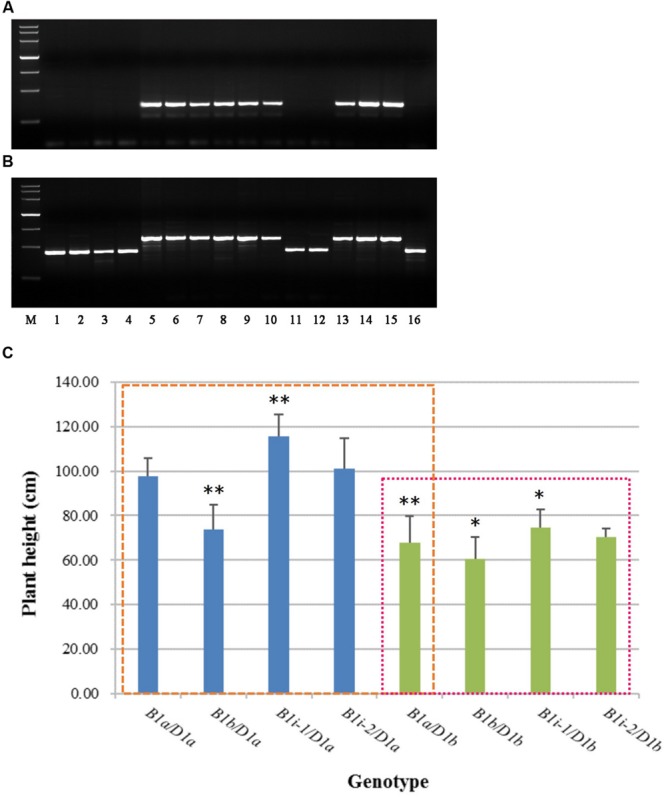
***Rht-B1i-1* significantly increases the plant height of wheat. (A)** PCR with B1i-MF1/MR1, **(B)** PCR with B1i-MF2/MR2, **(C)** Plant height in *Rht-B1i-1* and *Rht-B1i-2* genotypes of wheat. Lanes: *M*, molecular weight markers (DNA marker III, Tiangen Biotech Co. Ltd, China), the bands are 4,500, 3,000, 2,000, 1,200, 800, 500, and 200 bp, respectively; *1*, CS (*Rht-B1a*), *2*, Jinchun 3 (*Rht-B1b*), *3*, Mercia (*Rht-B1e*), *4*, Zhongyou 9507 (*Rht-B1h*), *5*, Guangtou (*Rht-B1i*), *6*, KaBka3 (*Rht-B1i*), *7*, Triumph (*Rht-B1i*), *8*, Dalibanmang (*Rht-B1i*), *9*, Huzhuhong (*Rht-B1i*), *10*, Dingxi 24 (*Rht-B1i*), *11*, Wumangchunmai (*Rht-B1i*), *12*, Kashi 1 (*Rht-B1i*), *13*, Xinshuguang 6 (*Rht-B1i*), *14*, Jinmai 56 (*Rht-B1i*), *15*, Jinan24 (*Rht-B1i*), *16*, Jimai 37 (*Rht-B1i*). The blue column represents *Rht-B1* allelic variations against the *Rht-D1a* background, and the green column represents *Rht-B1* allelic variations against the *Rht-D1b* background; the left box compares plant height for different genotypes using *B1a*/*D1a* as a control, and the right box compares plant height for different genotypes using *B1a*/*D1b* as a control. ^∗, ∗∗^Denotes significant differences at 5 and 1% probability levels, respectively.

To facilitate the use of *Rht-B1i* in wheat-breeding programs, and ascertain whether all the accessions containing *Rht-B1i* have 160 bp insertion in the promoter, we developed two allele-specific PCR markers (Supplementary Table S3), B1i-MF1/MR1 (dominant) and B1i-MF2/MR2 (codominant), based on the 160 bp insertion of *Rht-B1i.* The amplification products were 330 bp and 586 bp, respectively, in most *Rht-B1i* varieties (**Figure 3**). The PCR products of B1i-MF1/MR1 (**Figure 3A**) showed no amplification among the *Rht-B1* allelic variations *Rht-B1a, Rht-B1b, Rht-B1e*, and *Rht-B1h*, whereas nine of the 12 varieties carrying *Rht-B1i* produced a positive 330-bp band. The PCR products of B1i-MF2/MR2 (**Figure 3B**) displayed 426-bp bands from *Rht-B1a, Rht-B1b, Rht-B1e*, and *Rht-B1h*, whereas 586 bp bands were observed in nine of 12 varieties containing *Rht-B1i*. Hence, the primers B1i-MF1/MR1 and B1i-MF2/MR2 are allele-specific for most *Rht-B1i* accessions. However, there were three accessions (Wumangchunmai, Kashi 1, and Jimai 37) that contained *Rht-B1i* but could not be identified by these two markers. We therefore amplified and sequenced their promoters and found that they lacked a 160 bp insertion. Using B1i-MF1/MR1 and B1i-MF2/MR2 to screen 262 Chinese wheat MCC and 1,537 Chinese wheat-leading cultivars and important germplasms, we found 3.4 and 2.02% frequencies of *Rht-B1i*, respectively. This is lower than the 4.2 and 2.28%, respectively, reported by Li et al. (2013), indicating that the *Rht-B1i* genotype might have other haplotypes.

Therefore, we cloned and sequenced the promoters from 46 lines, which were validated as carrying *Rht-B1i* from 262 Chinese wheat MCC (11 lines) and 1,537 Chinese wheat-leading cultivars and important germplasms (35 lines). Of 11 Chinese wheat MCC, nine accessions had the 160 bp insertion; among 35 Chinese wheat-leading cultivars and important germplasms, 25 lines had the 160 bp insertion (Supplementary Table S2). Consequently, we subclassified the *Rht-B1i* genotype into *Rht-B1i-1* (75.8% had a 160 bp insertion in the promoter) and *Rht-B1i-2* (24.2% were missing a 160 bp insertion in the promoter). To identify the effect of the 160 bp insertion, we analyzed the plant height of *Rht-B1i-1* and *Rht-B1i-2* (**Figure 3C**). Whether under the background of *Rht-D1a* or *Rht-D1b, Rht-B1i-1* significantly increased plant height by 10.18 and 9.89% as compared with *Rht-B1a*, meanwhile, the height of *Rht-B1i-2* was increased, by 3.64 and 3.54%, with no significance, indicating that *Rht-B1i-1* significantly increased wheat plant height under both *Rht-D1a* and *Rht-D1b* backgrounds.

### The 160 bp Insertion Affect *Rht-B1i-1* Expression

**FIGURE 4:**
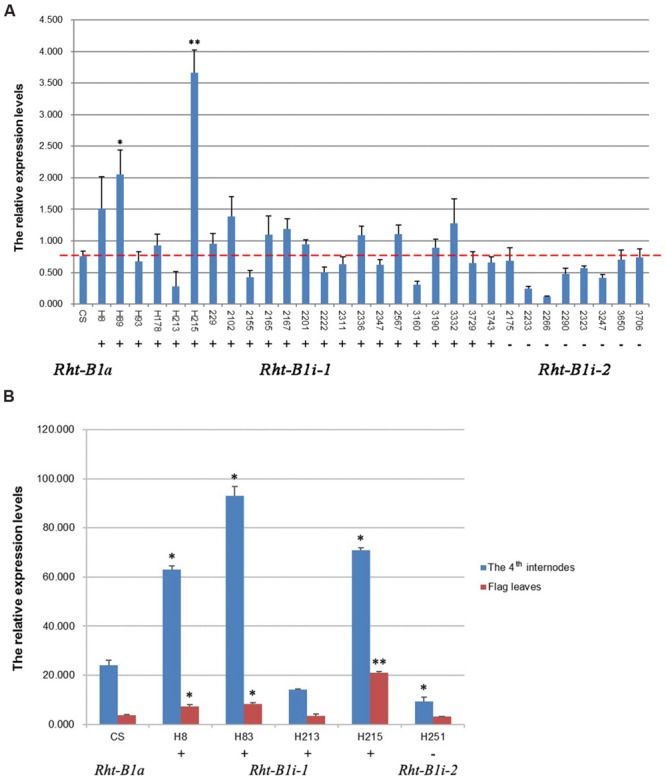
**Expression analysis of *Rht-B1i-1* and *Rht-B1i-2.* (A)** Expression analysis in seedlings, **(B)** Expression analysis in flag leaves and the fourth internodes. The red line shown the relative expression levels of *Rht-B1* in CS which containing *Rht-B1a* (control). ^∗, ∗∗^Denotes significant differences at 5 and 1% probability levels, respectively. +, – Denote *Rht-B1i-1* containing the 160 bp insertion and *Rht-B1i-2* missing the 160 bp insertion, respectively.

Structural analysis of promoters has wide implications in molecular biology, because it allows the prediction of gene expression profiles. We analyzed the GC content in the 1,500-bp section upstream from the translation initiation codon of the *Rht-B1* allelic variations and found that the AT-content of the four *Rht-B1* promoters (P1, 51.47%; P2, 52.53%; P3, 51.60%; P4, 51.80%) was greater than the corresponding GC content (48.53, 47.47, 48.40, and 48.20%, respectively), which is consistent with a role as an AT-rich plant gene-promoter element. To study the contribution of the 160 bp insertion to gene transcription, we performed quantitative real-time PCR (qRT-PCR) using the seedlings of CS and 30 other varieties containing *Rht-B1i*, flag leaves and the fourth internodes from four varieties of *Rht-B1i-1* genotype H8, H83, H213, H215, and one variety of *Rht-B1i-2* genotype H251 at the heading stage, and using *Ta4045* as the reference gene. For seedlings (**Figure 4A**), all eight varieties containing *Rht-B1i-2* (without the 160 bp insertion) expressed lower levels than wild-type *Rht-B1a*, but these differences were not statistically significant. Among the 22 varieties carrying *Rht-B1i-1* (with the 160 bp insertion), 13 expressed higher levels than *Rht-B1a* (statistically significant in 2/13), whereas nine varieties expressed lower levels than *Rht-B1a*. This indicates that the 160 bp insertion can have differential effects on gene expression. For flag leaves (**Figure 4B**), the variety containing *Rht-B1i-2* also expressed lower level than *Rht-B1a*, whereas three of the four varieties containing *Rht-B1i-1* expressed significantly higher levels than *Rht-B1a*. For the fourth internodes (**Figure 4B**), the expression level of the variety containing *Rht-B1i-2* was significantly lower than *Rht-B1a*, however, the expression levels of three varieties containing *Rht-B1i-1* were significantly higher than *Rht-B1a*. Overall, the 160 bp insertion can affect the expression of *Rht-B1i-1*.

### The Height-Increasing Effect of *Rht-B1i-1* May Be Related to Auxin

We carried out predictive analysis of the *cis*-acting elements in the promoters of *Rht-B1a* and *Rht-B1i-1* using PLACE^1^ and PlantCARE^2^. The analysis revealed various possible *cis*-acting elements in the two promoters: in addition to basic *cis*-regulatory elements, such as TATA-box, CAAT-box, and so on, there were many possible *cis*-acting elements that were related to phyto-hormone response, tissue-specific expression, and stress induction. This suggests that *Rht-B1* may participate in the regulation of multiple phyto-hormones and environmental signaling pathways. There were 453 and 485 possible *cis*-acting elements in the promoters of *Rht-B1a* and *Rht-B1i-1*, respectively, with the 32 additional possible *cis*-acting elements located in the 160 bp insertion in the promoter of *Rht-B1i-1* (**Table 1**). Among these 32 additional elements, the promoter of *Rht-B1i-1* contained four unique *cis*-acting elements, including an auxin response element (AUXREPSIAA4). This indicates that the regulatory network of *Rht-B1i-1* is highly complex and that the height-increasing effect of *Rht-B1i-1* may be related to auxin.

**Table 1:** *Cis*-acting elements analysis of the promoter of *Rht-B1a* and *Rht-B1i.*

Name	Description	*Rht-B1a*	*Rht-B1i-1*	*Rht-B1i-2*
ARR1AT	ARR1-binding element	22	25	22
AUXREPSIAA4	Auxin responsive element	0	1	0
INRNTPSADB	Light-responsive element	1	2	1
MYB1AT	MYB recognition site	0	1	0
MYB2CONSENSUSAT	MYB recognition site	2	3	2
EECCRCAH1	MYB binding site	1	2	1
MYB2AT	MYB binding site	1	2	1
MYBATRD22	MYB binding site	0	1	0
MYBCORE	MYB binding site	4	5	4
CACTFTPPCA1	Required for mesophyll expression	22	26	22
DOFCOREZM	Core site required for binding of Dof proteins	13	15	13
NODCON2GM	Putative nodulin consensus sequences	5	6	5
ROOTMOTIFTAPOX1	Motif found both in promoters of rolD	5	6	5
RHERPATEXPA7	Root Hair-specific *cis*-elements	8	9	8
RYREPEATBNNAPA	Required for seed specific expression	3	5	3
RYREPEATGMGY2	Required for seed specific expression	2	3	2
RYREPEATLEGUMINBOX	Required for seed specific expression	2	4	2
ANAERO2CONSENSUS	Involved in the fermentative pathway	2	3	2
CAATBOX1	CAAT promoter consensus sequence	13	14	13
CURECORECR	Copper-response element	18	20	18
GATABOX	Required for high level, light regulated, and tissue specific expression	19	20	19
OSE2ROOTNODULE	Organ-specific elements activated in infected cells of root nodules	5	6	5
TATABOXOSPAL	Binding site for OsTBP2	0	1	0

**FIGURE 5:**
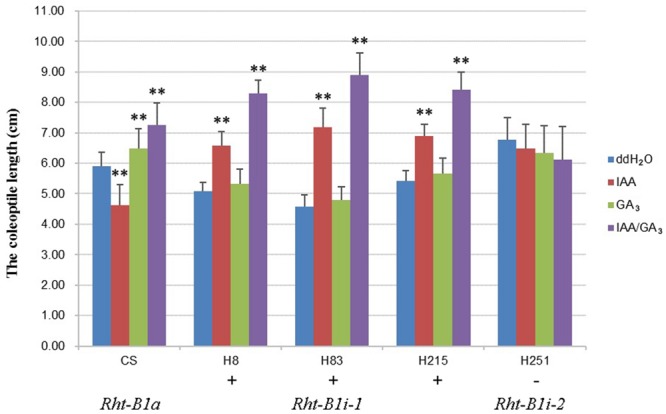
**The coleoptile length of wheat varieties with *Rht-B1i-1* and *Rht-B1i-2*.** The phyto-hormone treatments of three genotypes, *Rht-B1a, Rht-B1i-1*, and *Rht-B1i-2*, with ddH_2_O as a control. ^∗∗^Denote significant differences at the 1% probability level. +, – Denote *Rht-B1i-1* containing the 160 bp insertion and *Rht-B1i-2* missing the 160 bp insertion, respectively.

To study the effects of the 160 bp insertion on the function of *Rht-B1i*, we treated CS, three varieties containing *Rht-B1i-1* (H8, H83, and H215 which had significantly higher expression levels), and one variety containing *Rht-B1i-2* (H251) with IAA, GA_3_, and IAA/GA_3_ (**Figure 5**). The coleoptile length of CS carrying *Rht-B1a* was significantly reduced after IAA treatment, but it clearly increased after GA_3_ and IAA/GA_3_ treatment, indicating that *Rht-B1a* has a strong response to IAA, GA_3_, and IAA/GA_3_. However, we observed no significant difference in the coleoptile length of variety containing *Rht-B1i-2* after IAA, GA_3_, or IAA/GA_3_ treatment, indicating that *Rht-B1i-2* was insensitive to IAA, GA_3_, and IAA/GA_3_. The coleoptile length of varieties containing *Rht-B1i-1* showed no significant difference after GA_3_ treatment, but it significantly increased after IAA and, particularly, IAA/GA_3_ treatment. This suggests that *Rht-B1i-1* is also insensitive to GA_3_ and that the height-increasing effect of *Rht-B1i-1* might be caused by the synergistic action of IAA and GA_3_.

### The Promoter of *Rht-B1i-1* Plays a Role in the Response to GA_3_ and IAA

**FIGURE 6:**
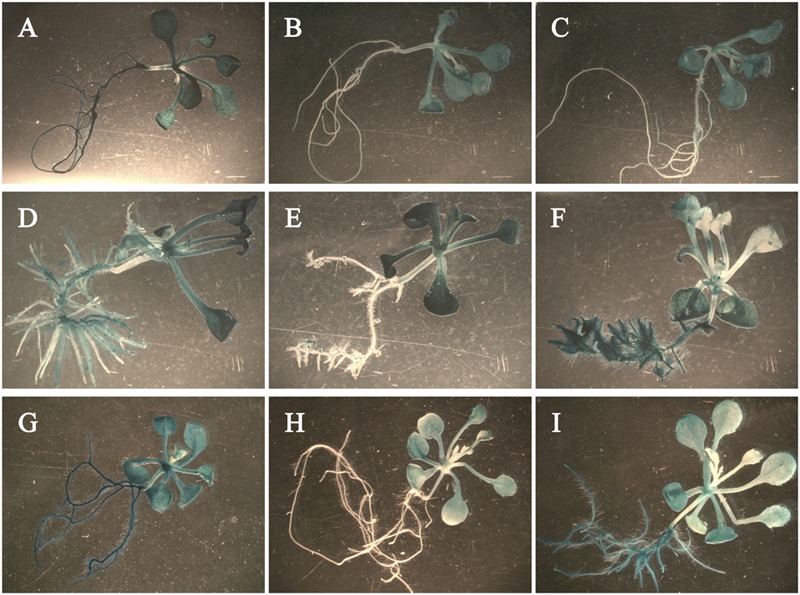
**The histochemical staining of phyto-hormone-treated transgenic *Arabidopsis* seedlings.** *pB1a* represents promoter of *Rht-B1a, pB1i-1* represents promoter of *Rht-B1i-1*. **(A–C)** *Arabidopsis* seedlings treated with GA_3_: **(A)** *35S::GUS* transgenic *Arabidopsis* seedling, **(B)** *pB1a::GUS* transgenic *Arabidopsis* seedling, **(C)** *pB1i-1::GUS* transgenic *Arabidopsis* seedling. **(D–F)** *Arabidopsis* seedlings treated with IAA: **(D)** *35S::GUS* transgenic *Arabidopsis* seedling, **(E)** *pB1a::GUS* transgenic *Arabidopsis* seedling, **(F)** *pB1i-1::GUS* transgenic *Arabidopsis* seedling. **(G–I)** *Arabidopsis* seedlings treated with IAA/GA_3_: **(G)** *35S::GUS* transgenic *Arabidopsis* seedling, **(H)** *pB1a::GUS* transgenic *Arabidopsis* seedling, **(I)** *pB1i-1::GUS* transgenic *Arabidopsis* seedling.

To study the response of the promoters to exogenous IAA and GA_3_, we fused the promoters of *Rht-B1a* (*pB1a*, 2,040 bp) and *Rht-B1i-1* (*pB1i-1*, 2,200 bp) with the *GUS* (β-glucuronidase) reporter gene using the binary vector pCAMBIA 3301-GUS. Vectors were then transformed into *Arabidopsis* ecotype Col-0. The transgenic *Arabidopsis* seedlings treated with GA_3_ had slender roots (**Figures 6A–C**), those treated with IAA had more lateral roots (**Figures 6D–F**), and those treated with IAA/GA_3_ had slender lateral roots (**Figures 6G–I**). The *35S::GUS* transgenic *Arabidopsis* seedlings expressed strong GUS activity in the entire plant after GA_3_ (**Figure 6A**), IAA (**Figure 6D**), and IAA/GA_3_ (**Figure 6G**) treatment. After GA_3_ treatment, the *pB1a::GUS* (**Figure 6B**) and *pB1i-1::GUS* (**Figure 6C**) transgenic *Arabidopsis* seedlings both expressed strong GUS activity in leaves and stems and weak GUS activity in roots, although the difference was non-significant. After treated with IAA and IAA/GA_3_, the *pB1a::GUS* transgenic *Arabidopsis* seedlings (**Figures 6E,H**) primarily expressed GUS activity in leaves and stems. However, the *pB1i-1::GUS* transgenic *Arabidopsis* seedlings (**Figures 6F,I**) strongly expressed GUS activity not only in leaves and stems but also in roots, illustrating that the promoter of *Rht-B1i-1* played a role in the response to IAA and IAA/GA_3_. The above results are in accordance with the predictive analysis of *cis*-acting elements in promoters and with the coleoptile lengths of wheat varieties treated with IAA, GA_3_, and IAA/GA_3_, demonstrating that the height-increasing effect of *Rht-B1i-1* may be caused by the synergistic effects of IAA and GA_3_.

## Discussion

The primary function of the *Rht-1* gene is to reduce the plant height of wheat. Börner et al. (1996) found that the relative strength of this effect among alleles at *Rht-1* loci is *Rht-B1a* < *Rht-B1d* < *Rht-B1b* < *Rht-B1e* < *Rht-B1c*, and *Rht-D1a* < *Rht-D1b* < *Rht-D1d* < *Rht-D1c*. Among these *Rht* alleles, the two ‘green revolution’ genes, *Rht-B1b* and *Rht-D1b*, are the most economically important and, by far, the most commonly used. Therefore we analyzed the effects of *Rht-B1b* on the plant height of wheat against the genetic backgrounds of *Rht-D1a* and *Rht-D1b* using 2 years (2012, 2013) of agronomic traits data. We quantified the effect in terms of the percentage reduction in plant height after the introduction of the *Rht-1* gene. Compared with the wild-type gene *Rht-B1a, Rht-B1b* significantly reduced plant height by 24.27 and 11.68% against the backgrounds of *Rht-D1a* and *Rht-D1b*, respectively. *Rht-B1b*+*Rht-D1b* reduced plant height by a statistically significant 38%. Flintham et al. (1997) also reported that *Rht-B1b* has the ability to reduce plant height by 14% but that *Rht-B1b* + *Rht-D1b* can reduce plant height by 42%, as can two copies of either *Rht-B1b* or *Rht-D1b* against near-isogenic backgrounds. Current studies are focusing on alleles with dwarfing effects; *Rht-B1b* and *Rht-D1b* are also associated with increased harvestable yield under favorable conditions (Gale and Youssefian, 1985; Flintham et al., 1997; Chapman et al., 2007). However, *Rht-B1b* and *Rht-D1b* also contribute to reduced coleoptile length and seedling vigor (Rebetzke et al., 2007; Addisu et al., 2009) and may reduce crop water-use efficiency in unfavorable environments (Butler et al., 2005; Chapman et al., 2007).

The discovery of new beneficial *Rht-1* alleles is very important for wheat-breeding programs. Our previous study (Li et al., 2013) identified six new *Rht-A1* allelic variations (*Rht-A1b*–*g*), eight new *Rht-B1* allelic variations (*Rht-B1h*–*o*), and six new *Rht-D1* allelic variations (*Rht-D1e*–*j*) by a modified EcoTILLING method. Among these, *Rht-B1h, Rht-B1i*, and *Rht-B1j* have relatively high frequencies in Chinese wheat MCC and Chinese wheat-leading cultivars and important germplasms. Interestingly, *Rht-B1i* containing a single missense SNP (A614G) in the coding region significantly increased wheat plant height against the background of both *Rht-D1a* (11.68%) and *Rht-D1b* (7.89%), suggesting that *Rht-B1i* could counteract the effects of *Rht-D1b*. Elucidating the height-increasing effect of *Rht-B1i* will contribute to our understanding of the regulatory mechanism of *Rht-1* and aid in the use of *Rht-B1i* for ameliorating excessive dwarfism in wheat.

The missense SNP occurring in *Rht-B1i* results in an E205G amino acid change in the Poly S/T/V region of the encoded DELLA protein. However, the SIFT score (0.41) indicates that the mutation has no obvious effect on the protein (Li et al., 2013), suggesting that the height-increasing effect does not result directly from the missense mutation in the coding region. The expression of a protein depends on the transcription induction of the corresponding gene; therefore, the analysis of promoter regions is highly relevant. Li et al. (2011) found that base mutants in the pyrimidine box upstream of the *BnGID1* sequence probably explain the low expression level of the GID1 protein. Similarly, Park et al. (2013) showed that the DELLA and BOI proteins inhibit GA responses by binding to the promoters of GA-responsive genes. Recognizing the importance of these regions, we isolated the promoters of *Rht-B1* alleles, found a 160 bp insertion, and classified *Rht-B1i* into *Rht-B1i-1* (with the 160 bp insertion in the promoter) and *Rht-B1i-2* (without the 160 bp insertion in the promoter) genotypes. Wilhelm et al. (2013b) directly sequenced the ORF and 5′ and 3′ flanking regions in western bread cultivars and tetraploid and diploid wheat and also identified three haplotypes (*Rht-B1a_2, 3, 4*) that have a 160 bp insertion in the 5′ flanking regions. Sequence alignment revealed several variants of *Rht-B1i: Rht-B1a_2, Rht-B1a_3*, and *Rht-B1a_4*. *Rht-B1a_2* is identical to *Rht-B1i-1*; *Rht-B1a_3* has two SNPs in the 5′ flanking region; and *Rht-B1a_4* has a CTA insertion in the 3′ flanking region. The *Rht-B1* 160 bp insertion is also present in a high frequency in western wheat accessions, and has a wide geographic distribution, suggesting that they are prevalent in modern bread wheat varieties. But the 160 insertion was not identified in any tetraploid and diploid wheat accessions, indicating that it is a recent event. In addition, the 160 bp insertion occurs in the middle of a highly conserved non-coding sequences among three wheat *Rht-1* homoeologs and in *Rht-1 Poaceae* orthologs, could be important *Rht-1* regulatory regions (Wilhelm et al., 2013a,b).

*Rht-B1i-1* with the 160 bp insertion in the promoter significantly increases the plant height of wheat against both the *Rht-D1a* and *Rht-D1b* backgrounds. To study the contribution of the 160 bp insertion to gene transcription, we analyzed the expression profiles of *Rht-B1i-1* and *Rht-B1i-2* with *Rht-B1a* as a control. qRT-PCR analysis of CS and varieties containing *Rht-B1i* at the seedling and heading stage indicated that the 160 bp insertion might affect gene expression. At the seedling stage, nine of 22 varieties containing *Rht-B1i-1* and all eight varieties containing *Rht-B1i-2* expressed lower levels than wild-type *Rht-B1a*, whereas other 13 of 22 varieties carrying *Rht-B1i-1* expressed higher levels than *Rht-B1a* (statistically significant in 2/13). Furthermore, at the heading stage, the variety H251 containing *Rht-B1i-2* expressed significant lower level than *Rht-B1a* in the fourth internodes, whereas, the expression levels of three varieties H8, H83, and H215 containing *Rht-B1i-1* were significantly higher than *Rht-B1a* in flag leaves and the fourth internodes. Although the *Rht-B1i* gene was expressed at different levels in different varieties at seedling and heading stage, the general trend is relatively consistent, indicating the 160 bp insertion is likely to promote the transcription of the *Rht-B1i-1* gene. Compared with the three spring wheat varieties H8, H83, and H215, the expression levels of *Rht-B1i-1* in H213 (which is a weak winter wheat variety from Qinghai province, China) were lower than that of CS at seedling and heading stage. Their difference in the expression levels of *Rht-B1i-1* may be caused by the genetic backgrounds, this needs further investigation. Wilhelm et al. (2013b) reported that the insertions may have a minor effect on *Rht-B1* transcript abundance in seedling tissue, although the normalized *Rht-B1* transcript levels were slightly (non-significantly) reduced. The discrepancy among these varieties may be explained by their different genetic backgrounds, and Wilhelm et al. (2013b) used only two lines with the 160 bp insertion (‘Mercia’ and ‘Paragon’) for qRT-PCR. Moreover, the *Rht-1* gene was expressed at different levels in different tissues and developmental stages (Pearce et al., 2011), our previous study showed that *Rht-1* preferentially expressed in the fourth internodes than flag leaves at the heading stage (unpublished data). The effect of the 160 bp insertion on transcript needs more analysis in more tissues and developmental stages.

Wilhelm et al. (2013a) found that *Rht-B1a-160* was associated with a small reduction in GA sensitivity relative to *Rht-B1a-0* accessions, but the difference was not statistically significant. In the present study, the coleoptile lengths of varieties containing *Rht-B1i-1* and *Rht-B1i-2* showed no significant effect of GA_3_ treatment, indicating that *Rht-B1i* was insensitive to GA_3_. *Rht-B1b* and *Rht-D1b* contain a single nucleotide change introducing a premature stop codon in the DELLA domain of encoded proteins which conferred dwarfism and GA insensitivity, as well as *Rht-B1d, Rht-B1e*, and *Rht-D1d* (Peng et al., 1999; Hedden, 2003; Pearce et al., 2011; Li A.X. et al., 2012; Li et al., 2013). It is very surprise that *Rht-B1i-1* who could increases the plant height of wheat was insensitive to GA_3_, the mechanism needs further investigation. Since the promoter of *Rht-B1i-2* is the same as *Rht-B1a*, while their response to GA_3_ are different, suggesting the missense SNP occurring in *Rht-B1i* resulted in an E205G amino acid change in the Poly S/T/V region has an effect on the activity of Rht-B1i protein. Itoh et al. (2002) revealed that the polyS/T/V region had a strong suppression function for the activity of SLR protein. The function of this missense SNP in *Rht-B1i* needs further investigation.

*Rht-B1* may participate in the regulation of multiple phyto-hormones and environmental signaling pathways because of the various *cis*-acting elements in its promoters. The 160 bp insertion in the promoter of *Rht-B1i-1* contained 32 possible *cis*-acting elements not found in *Rht-B1a*. Among these 32 elements, four, including the auxin response element AUXREPSIAA4, are unique to *Rht-B1i-1.* This indicates that the height-increasing effect of *Rht-B1i-1* may be related to auxin. GA can promote the transport of auxin, whereas auxin can promote the biosynthesis of GA (Frigerio et al., 2006). In root growth and differentiation, GA and DELLA proteins control the transport of auxin and coordinate cell division and differentiation (Moubayidin et al., 2010). In the present study, the coleoptile lengths of wheat varieties with the *Rht-B1i-1* genotype responded to IAA, and IAA/GA_3_, while that of *Rht-B1i-2* genotype did not respond to IAA, and IAA/GA_3_, and the transgenic *Arabidopsis* seedlings with promoter of *Rht-B1i-1* also responded to IAA, and IAA/GA_3_, that of *Rht-B1i-2* did not, illustrating the height-increasing effect of *Rht-B1i-1* may be caused by the synergistic effects of IAA and GA_3_. Further comprehensive study should be launch to elucidate the mechanism of the height-increasing effect for *Rht-B1i-1*.

## Author Contributions

XYL, XL, AL, MP, and WY participated in experiments, drafting the manuscript and proposal writing. DL and JS participated in material preparation, AZ and WY participated in experimental design and corrected the manuscript. MS corrected the manuscript.

## Conflict of Interest Statement

The authors declare that the research was conducted in the absence of any commercial or financial relationships that could be construed as a potential conflict of interest.

## Footnotes

1 http://www.dna.affrc.go.jp/PLACE/signalscan.html

2 http://bioinformatics.psb.ugent.be/webtools/plantcare/html/
