# Supplementary material for: The 160 bp Insertion in the Promoter of Rht-B1i Plays a Vital Role in Increasing Wheat Height
Source: Front Plant Sci. 2016 Mar 16;7:307. doi: 10.3389/fpls.2016.00307 (PMC4792873; doi:10.3389/fpls.2016.00307)
Supplement: Supplementary file 1 [file Table_1.DOCX]

**Table S1 The seventy wheat germplasms with different *Rht-B1* allelic variations**

| *Rht-B1* | No. | Accession | Code | Cultivation region^a^ | Category^b^ | Promoter type^c^ | Promoter length |
| --- | --- | --- | --- | --- | --- | --- | --- |
| *B1a* | H260 | Chinese spring | ZM005452 |  | B | P0 | 2040 bp |
| *B1a* | H16 | Niuzhijia | ZM001259 | I | L | P1 | 2040 bp |
| *B1a* | H76 | Anhui 3 | ZM010261 | III | B | P1 | 2040 bp |
| *B1a* | H127 | Youbao | ZM009411 | II | B | P1 | 2040 bp |
| *B1a* | H135 | Xiannong 39 | ZM017208 | II | B | P1 | 2040 bp |
| *B1a* | H136 | Jinan 17 | Jinan17 | II | B | P1 | 2040 bp |
| *B1a* | H145 | Huangguaxian | ZM003050 | II | L | P1 | 2040 bp |
| *B1a* | H195 | Shanmai | ZM020774 | VIII | L | P1 | 2040 bp |
| *B1a* | H200 | Shanmai | ZM020770 | VIII | L | P1 | 2040 bp |
| *B1a* | H262 | Yanzhan 1 | Yanzhan1 | II | B | P1 | 2040 bp |
| *B1b* | H3 | Jinchun 3 | ZM014440 | VII | B | P1 | 2040 bp |
| *B1b* | H73 | Yangmai 158 | H01094 | III | B | P1 | 2040 bp |
| *B1b* | H86 | Kelao 4 | ZM014682 | VI | B | P1 | 2040 bp |
| *B1b* | H94 | St 2422/464 | MY002776 | Foreign | I | P1 | 2040 bp |
| *B1b* | H97 | Norin 10 | MY000054 | Foreign | I | P1 | 2040 bp |
| *B1b* | H98 | Suwon 86 |  | Foreign | I | P1 | 2040 bp |
| *B1b* | H137 | Xiaoyan 6 | ZM017079 | II | B | P1 | 2040 bp |
| *B1b* | H138 | Shannong 7859 | ZM017231 | II | B | P1 | 2040 bp |
| *B1b* | H143 | Zhengzhou 741 | ZM015988 | II | B | P1 | 2040 bp |
| *B1b* | H204 | Hongmangmai | ZM020720 | VIII | L | P1 | 2040 bp |
| *B1b* | H210 | Gaoyuan 506 | ZM010116 | VIII | B | P1 | 2040 bp |
| *B1b* | H212 | Ningchun 4 | ZM017424 | VIII | B | P1 | 2040 bp |
| *B1b* | H219 | Bimai 26 | ZM023312 | IV | B | P1 | 2040 bp |
| *B1b* | H222 | Xingyi 4 | ZM023315 | IV | B | P1 | 2040 bp |
| *B1e* |  | Mercia-*B1e* |  | Foreign |  | P1 | 2040 bp |
| *B1h* | H39 | Yuandong 822 | ZM013548 | I | B | P2 | 2237 bp |
| *B1h* | H106 | Atlas 66 | MY000295 | Foreign | I | P1 | 2040 bp |
| *B1h* | H128 | Bainong 3217 | ZM017936 | II | B | P2 | 2237 bp |
| *B1h* | H132 | Jimai 2 | ZM009126 | II | B | P2 | 2237 bp |
| *B1h* | H139 | Aifeng 3 | ZM009603 | II | B | P2 | 2237 bp |
| *B1h* | H172 | Baimangmai | ZM003650 | II | L | P2 | 2237 bp |
| *B1h* | H192 | Zangdong 4 | ZM010580 | IX | B | P2 | 2237 bp |
| *B1h* | H220 | Guinong 10 | ZM023371 | IV | B | P2 | 2237 bp |
| *B1h* | H261 | Zhengmai 9023 | Zhengmai9023 | II | B | P1 | 2040 bp |
| *B1i* | H8 | Dabaipi | ZM017481 | VII | L | P3 | 2200 bp |
| *B1i* | H83 | Guangtou | ZM004338 | VI | L | P3 | 2200 bp |
| *B1i* | H87 | Xinshuguang 1 | ZM009657 | VI | B | P3 | 2200 bp |
| *B1i* | H89 | Xinshuguang 6 | ZM009662 | VI | B | P3 | 2200 bp |
| *B1i* | H93 | Кавказ |  | Foreign | I | P3 | 2200 bp |
| *B1i* | H101 | Triumph | MY002966 | Foreign | I | P3 | 2200 bp |
| *B1i* | H178 | Dalibanmang | ZM001742 | II | L | P3 | 2200 bp |
| *B1i* | H213 | Huzhuhong | ZM017354 | VIII | B | P3 | 2200 bp |
| *B1i* | H215 | Dingxi 24 | ZM009893 | VIII | B | P3 | 2200 bp |
| *B1i* | H251 | Wumangchunmai | ZM005336 | X | L | P1 | 2040 bp |
| *B1i* | H257 | Kashi 1 | H02027 | X | B | P1 | 2040 bp |
| *B1j* | H14 | Dahongmai | ZM010600 | VII | L | P1 | 2040 bp |
| *B1j* | H69 | Paozimai | ZM007298 | III | L | P1 | 2040 bp |
| *B1j* | H74 | Enmai 4 | ZM016244 | III | B | P1 | 2040 bp |
| *B1j* | H75 | Emai 6 | ZM010314 | III | B | P1 | 2040 bp |
| *B1j* | H85 | Kefeng 3 | ZM014679 | VI | B | P1 | 2040 bp |
| *B1j* | H95 | Nanda 2419 | ZM010176 | Foreign | I | P1 | 2040 bp |
| *B1j* | H96 | Orofen | MY002255 | Foreign | I | P1 | 2040 bp |
| *B1j* | H134 | Zhengzhou 6 | ZM009463 | II | B | P1 | 2040 bp |
| *B1j* | H209 | Ganmai 8 | ZM009803 | VIII | B | P1 | 2040 bp |
| *B1j* | H211 | Qingchun 28 | ZM017383 | VIII | B | P1 | 2040 bp |
| *B1j* | H217 | Shuwan 8 | ZM010490 | IV | B | P1 | 2040 bp |
| *B1j* | H221 | Yunmai 34 | ZM016965 | IV | B | P1 | 2040 bp |
| *B1j* | H228 | Jiangmai | ZM011774 | IV | L | P1 | 2040 bp |
| *B1k* | H5 | Bihongsui | ZM009772 | VII | B | P1 | 2040 bp |
| *B1k* | H180 | Gejiaxiang | ZM012971 | IX | L | P1 | 2040 bp |
| *B1k* | H198 | Galaohan | ZM005105 | VIII | L | P1 | 2040 bp |
| *B1k* | H202 | Baidatou | ZM004862 | VIII | L | P1 | 2040 bp |
| *B1k* | H247 | Hongchunmai | ZM005294 | X | L | P1 | 2040 bp |
| *B1k* | H253 | Hongjinbaoyin | ZM013034 | X | L | P1 | 2040 bp |
| *B1l* | H224 | Tongjiabaxiaomai | ZM007916 | IV | L | P4 | 2020 bp |
| *B1l* | H226 | Baimaizi | ZM008547 | IV | L | P4 | 2020 bp |
| *B1m* | H125 | Taishan 1 | ZM009405 | II | B | P1 | 2040 bp |
| *B1m* | H164 | Meiqianwu | ZM006160 | II | L | P1 | 2040 bp |
| *B1n* | H81 | Huoqiu | ZM004433 | VI | L | P1 | 2040 bp |
| *B1o* | H111 | Shengen | ZM007521 | V | L | P1 | 2040 bp |

^a^ I, Northern winter wheat region; II, Yellow and Huai River Valley winter wheat region; III, Middle and Low Yangtze Valley winter wheat region; IV, Southwestern winter wheat region; V, Southern winter wheat region; VI, Northeastern spring wheat region; VII,Northern spring wheat region; VIII, Northwestern spring wheat region; IX, Qing-Tibetan Plateau winter-spring wheat region; X, Xinjiang winter-spring wheat region;

^b^ L, represents Chinese wheat landraces; B, represents Chinese bred cultivars; I, represents introduced wheat varieties from foreign countries.

^c^P0, the promoter of *Rht-B1a*(FR719732); P1, the promoter which was exactly coincide exactly with P0; P2, the promoter which containingseven SNPs and a 197 bp insertion at 596 bp upstream of the start codon compared to P0; P3, the promoter which carrying a 160 bp insertion at 365 bp upstream of the start codon by contrast with P0; P4, the promoter who had a 20 bp deletion at 3 bp upstream of the start codon by comparison with P0.

Table S2 The thirty-five wheat germplasms with *Rht-B1i* in Chinese wheat leading cultivars and important germplasms

| No. | Accession | Promoter type^a^ | 160 bp insertion | Promoter length | Genotype |
| --- | --- | --- | --- | --- | --- |
| 229 | PBM34 | P3 | Yes | 2200 bp | *Rht-B1i-1* |
| 677 | 97-149-11-1 | P3 | Yes | 2200 bp | *Rht-B1i-1* |
| 1425 | Jinnong 211 | P3 | Yes | 2200 bp | *Rht-B1i-1* |
| 1488 | Deguo 8661 wan | P3 | Yes | 2200 bp | *Rht-B1i-1* |
| 1667 | Ai 5 | P3 | Yes | 2200 bp | *Rht-B1i-1* |
| 2042 | Jinmai 56 | P3 | Yes | 2200 bp | *Rht-B1i-1* |
| 2074 | ARBOLA | P3 | Yes | 2200 bp | *Rht-B1i-1* |
| 2075 | MLADKA | P3 | Yes | 2200 bp | *Rht-B1i-1* |
| 2082 | PETRANA | P3 | Yes | 2200 bp | *Rht-B1i-1* |
| 2085 | PAULINA | P3 | Yes | 2200 bp | *Rht-B1i-1* |
| 2102 | VANDA | P3 | Yes | 2200 bp | *Rht-B1i-1* |
| 2155 | 10-2 dali | P3 | Yes | 2200 bp | *Rht-B1i-1* |
| 2165 | Wen 6-986 | P3 | Yes | 2200 bp | *Rht-B1i-1* |
| 2167 | Lu 03-17 | P3 | Yes | 2200 bp | *Rht-B1i-1* |
| 2201 | Lu 03-42 | P3 | Yes | 2200 bp | *Rht-B1i-1* |
| 2222 | Linyou 359 | P3 | Yes | 2200 bp | *Rht-B1i-1* |
| 2311 |  | P3 | Yes | 2200 bp | *Rht-B1i-1* |
| 2336 |  | P3 | Yes | 2200 bp | *Rht-B1i-1* |
| 2347 |  | P3 | Yes | 2200 bp | *Rht-B1i-1* |
| 2567 | Zaoyou 504 | P3 | Yes | 2200 bp | *Rht-B1i-1* |
| 3160 | Xichangza 852 | P3 | Yes | 2200 bp | *Rht-B1i-1* |
| 3190 | Chuan P16 | P3 | Yes | 2200 bp | *Rht-B1i-1* |
| 3332 | 04G238-1 | P3 | Yes | 2200 bp | *Rht-B1i-1* |
| 3729 | 9814B | P3 | Yes | 2200 bp | *Rht-B1i-1* |
| 3743 | 97-141 | P3 | Yes | 2200 bp | *Rht-B1i-1* |
| 1192 | 2913297 | P1 | No | 2040 bp | *Rht-B1i-2* |
| 2084 | ILONA | P1 | No | 2040 bp | *Rht-B1i-2* |
| 2175 | Zhoumai 17 | P1 | No | 2040 bp | *Rht-B1i-2* |
| 2233 | 98112 | P1 | No | 2040 bp | *Rht-B1i-2* |
| 2266 |  | P1 | No | 2040 bp | *Rht-B1i-2* |
| 2290 |  | P1 | No | 2040 bp | *Rht-B1i-2* |
| 2323 |  | P1 | No | 2040 bp | *Rht-B1i-2* |
| 3247 |  | P1 | No | 2040 bp | *Rht-B1i-2* |
| 3650 | Xinkang 5 | P1 | No | 2040 bp | *Rht-B1i-2* |
| 3706 | Jimai 37 | P1 | No | 2040 bp | *Rht-B1i-2* |

^a^P1, the promoter which was exactly coincide exactly with the promoter of *Rht-B1a* (FR719732); P3, the promoter which carrying a 160 bp insertion at 365 bp upstream of the start codon by contrast with the promoter of *Rht-B1a*.

**Table S3 Primers used in this study**

| Name | Sequence (5’ to 3’) | Purpose |
| --- | --- | --- |
| PB-CF | GCTCGCATCACCAAAGTATCC | Cloning for promoter of *Rht-B1* |
| PB-CR | GGTACTCGCGCTTCATGAT |  |
| B1i-MF1 | CAGACGATATTTAACTGGCCGATTGA | PCR marker for *Rht-B1i* |
| B1i-MR1 | GGGAGCGGCAGCGTAGTAGTTGTA |  |
| B1i-MF2 | CTCTAATTTGCGGGGATTTC |  |
| B1i-MR2 | CGTCCTGGTACTCGCGCTTCAT |  |
| Rht-B1.EF | GGTAGGGAGGCGAGAGGCGAG | qRT-PCR for *Rht-B1i* |
| Rht-B1.ER | CTCGGACGAGCCCATGCCT |  |
| Ta4045-EF | CCTGCCCCGTACAACCTTGAG |  |
| Ta4045-ER | CACCGTTGCGATAGTCCTGAAAC |  |
| PB-*Pst*I-F | CTGCAGCATCACCAAAGTATCCAACGTAC | Promoter analysis for *Rht-B1i* |
| PB-*Nco*I-R | CCATGGGGTACTCSCGCTTCAT |  |

F, forward primer; R, reverse primer; the underlined regions were the sites for restriction digest.
